# Antibiotics Knowledge and Prescription Patterns Among Dental Practitioners in Croatia, Bosnia and Herzegovina, and Serbia: A Comparative E-Survey with a Focus on Medically Healthy and Compromised Patients

**DOI:** 10.3390/antibiotics13111061

**Published:** 2024-11-08

**Authors:** Marija Badrov, Danijela Marovic, Antonija Tadin

**Affiliations:** 1 Department of Restorative Dental Medicine and Endodontics, Study of Dental Medicine, School of Medicine, University of Split, 21000 Split, Croatia; marija.badrov@mefst.hr (M.B.); atadin@mefst.hr (A.T.); 2 Department of Endodontics and Restorative Dental Medicine, University of Zagreb School of Dental Medicine, 10000 Zagreb, Croatia; 3 Department of Maxillofacial Surgery, Clinical Hospital Centre Split, 21000 Split, Croatia

**Keywords:** antibiotics, antimicrobial resistance, dentistry, dentist, knowledge, prescription practices, Croatia, Bosnia and Herzegovina, Serbia

## Abstract

Background: The non-specific prescription of antibiotics, especially in dentistry, contributes to the global problem of antimicrobial resistance and highlights the need for education on the proper use and serious consequences of overprescribing these drugs. The main objective of this study is to assess and evaluate antibiotic knowledge and prescribing patterns in dental practice in Croatia, Bosnia and Herzegovina, and Serbia, focusing on understanding the rationale for prescribing, adherence to evidence-based guidelines, and dentists’ awareness of antibiotic resistance. Methods: A total of 795 dentists participated in this electronic cross-sectional survey (Croatia N = 336, Bosnia and Herzegovina N = 176, and Serbia N = 283). The study utilized a self-structured questionnaire to collect data on various aspects of antibiotic use, including knowledge, prescribing practices, awareness of guidelines, and demographic and professional information about dentists. Data analysis included the Mann–Whitney test, the Kruskal–Wallis test with post hoc analysis, and chi-square tests, with statistical significance set at *p* < 0.05. Results: The overall score for the participants’ knowledge of antibiotics was 6.40 ± 1.40 out of a maximum of eight points, which indicates a generally good level of knowledge among dentists. Factors such as gender, specialty, and practice location significantly influenced the level of knowledge (*p* < 0.05). However, actual prescribing practice was a cause for concern. Only 66.1% of Croatian dentists felt they had received adequate training during their studies, and even fewer in Serbia (48.4%) and Bosnia (46.6%). It is noteworthy that 9.7% of dentists in Bosnia and Herzegovina prescribe antibiotics at the request of patients, while 22.3% of Croatian and 25.4% of Serbian dentists do so. Many dentists prescribe no or only one antibiotic per week. In addition, 50.9% of Croatian dentists reported adverse effects related to the use of antibiotics, while only 31.3% of Bosnian and 33.6% of Serbian dentists reported similar experiences. Conclusions: While the study results indicate that dentists in the region generally possess good knowledge of antibiotic use, there are significant discrepancies between this knowledge and actual prescribing practices. This highlights the need for enhanced educational programs and awareness initiatives focused on proper antibiotic guidelines to improve prescribing behaviors.

## 1. Introduction

Antibiotics are crucial in dentistry to treat infections, prevent complications in high-risk patients, and support post-procedure recovery, mainly complementing endodontic, surgical, or periodontal treatments [1,2]. In healthy patients, antibiotics are reserved for acute infections like periapical abscesses or cellulitis with systemic symptoms such as fever, lymphadenopathy, or trismus. Prophylactic antibiotics may be required for medically compromised patients at higher risk, such as those with heart disease or weakened immunity [3,4,5]. Dental antibiotic guidelines stress accurate diagnosis prior to prescribing, advocating for targeted broad-spectrum antibiotics [6,7,8,9]. Treatment duration should be minimized to effectively control infection while reducing resistance and side effects. Amoxicillin is most commonly prescribed, with clindamycin and metronidazole as alternatives. Precise dosing ensures efficacy, shorter treatment times, better patient outcomes, and helps combat antibiotic resistance [2,3,4,5,9].

While antibiotics are beneficial, their overuse risks contributing to antibiotic resistance. Dentists and oral surgeons are responsible for about 10% of antibiotic prescriptions, often without adequate clinical justification [10,11]. Overprescribing, sometimes due to patient requests or caution, encourages resistant bacterial strains, undermining treatment efficacy. This issue stems from both dentist pressures and patient expectations, with patients often mistakenly believing antibiotics will hasten recovery [12,13]. Combating resistance requires dentists to follow evidence-based guidelines, assess antibiotic necessity, educate patients on proper use, favor narrow-spectrum antibiotics, minimize unnecessary prophylaxis, practice strict infection control, and stay updated on research while routinely reviewing their prescribing habits [5,14,15].

Numerous articles conducted worldwide, including in Croatia, Bosnia and Herzegovina, and Serbia, emphasize that non-compliance with recommended prescribing protocols—particularly in terms of the selection of appropriate antibiotics for suspected pathogens, site of infection, dosing regimens, and duration of therapy—poses a significant challenge to antibiotic stewardship in dentistry [16,17,18,19,20,21,22,23,24,25,26,27,28,29,30,31,32,33,34]. In Croatia, awareness of antimicrobial resistance among dentists is very high, but the overuse of antibiotics remains a major concern [27,34]. The use of antibiotics in dentistry has increased in parallel with the general increase in drug consumption, with a growth rate of over 6% in the ten years from 2014 to 2024 [24,25]. During this period, amoxicillin with clavulanic acid was the most frequently prescribed antibiotic, followed by amoxicillin, clindamycin, and metronidazole [25,26]. On average, each dental practice issued 133 antibiotic prescriptions per year [24], with around 30% being prescribed for clinical diagnoses that do not justify antibiotic treatment [25]. The main indications for the use of antibiotics include endodontic and oral surgical procedures [26,27,29,30]. In Serbia, research has shown that dentists are not sufficiently informed about dental ethics, which has a negative impact on their practice of prescribing antibiotics. This includes inappropriate behaviors such as prescribing antibiotics when they are not necessary, choosing broad-spectrum antibiotics without appropriate indication, and issuing prescriptions without thorough examination or for indications that are beyond their competence [31]. In contrast to the practices of Croatian dentists, amoxicillin is the first-choice antibiotic in Serbia, followed by clindamycin [32]. In a 2019 study conducted among dentists in Bosnia and Herzegovina, respondents noted that the use of antimicrobials is often excessive, unnecessary, and indiscriminate. They also agreed that antimicrobial resistance is a significant global problem [34]. As confirmed by studies in Croatia, antibiotics such as amoxicillin with clavulanic acid, and amoxicillin followed by clindamycin and metronidazole, are frequently used in endodontics in Bosnia and Herzegovina [34]. Although dentists in some studies report that they follow guidelines, it is important to emphasize that most dental societies of different specialties in Croatia, Bosnia and Herzegovina, and Serbia do not have guidelines available in their native language. Instead, they rely on recommendations from European and global dental organizations [8,28,33]. This underlines the need to standardize antibiotic guidelines in these societies, especially regarding the use in medically healthy and immunocompromised patients at the national level.

Given the challenges dentists face in using antibiotics—stemming from unfamiliarity with guidelines, inconsistencies, and workplace pressures—this study aims to assess antibiotic knowledge and prescribing patterns among dentists treating both healthy and immunocompromised patients in Croatia, Serbia, and Bosnia and Herzegovina. Addressing these issues is vital for effective patient care and antibiotic stewardship. The study also examines how socio-demographic and professional factors influence antibiotic knowledge, hypothesizing no significant differences among respondents from different countries.

## 2. Results

Table 1 presents demographic and professional data for the total number of respondents, as well as by country, along with the knowledge scores on antibiotics. A total of 795 respondents participated in the study, with a mean age of 37.20 ± 8.90 years (Md = 35.00; min = 24, max = 69). On average, dental practitioners prescribed 2.1 ± 2.1 antibiotic courses per week (Md = 2.00; minimum = 0, maximum = 20). Among them, 336 were dental practitioners from Croatia, with a mean age of 37.4 ± 10.1 years (Md = 34.00; min = 25, max = 69), who prescribed an average of 2.1 ± 2.1 antibiotics per week (Md = 2.00; min = 0, max = 20). From Bosnia and Herzegovina, 176 dentists with a mean age of 38.1 ± 10.6 years (Md = 38.00; min = 26, max = 61) typically prescribed 2.5 ± 2.8 antibiotics weekly (Md = 2.00; min = 0, max = 20). Meanwhile, 283 dentists from Serbia, with a mean age of 36.5 ± 7.4 years (Md = 35.00; min = 24, max = 53), prescribed an average of 1.8 ± 1.5 antibiotics per week (Md = 1.00; min 0, max 10).

The total antibiotics knowledge score among all participants was 6.40 ± 1.40 out of a maximum of eight (Md = 7.00; IQR 6.00–8.00, min = 0, max = 8). For Croatian participants, the score averaged 6.30 ± 1.43 (Md = 7.00; IQR 5.00–7.00, min = 0, max = 8). Bosnian participants had a mean score of 6.41 ± 1.51 (Md = 7.00; IQR 6.00–8.00, min = 2, max = 8), while Serbian participants scored 6.55 ± 1.39 (Md = 7.00; IQR 6.00–8.00, min = 2, max = 8). There was no statistically significant difference in knowledge scores among dental practitioners based on their country of practice (*p* = 0.093). Female participants demonstrated significantly better knowledge compared to males (*p* ≤ 0.001). Among different specialties, pediatric dentists had the highest knowledge scores compared to general dentists and other specialties (*p* = 0.002). Additionally, dentists working in urban areas showed better knowledge than those practicing in rural areas (*p* = 0.034)

Table 2 illustrates that most respondents rated their knowledge of antibiotics as moderate (N = 566, 71.2%). A total of 57.6% of Serbian practitioners prescribe none or only one antibiotic weekly, whereas nearly half of Croatian (49.1%) and Bosnian (48.9%) practitioners prescribe between 2 and 5 antibiotics weekly. Notably, 50.9% of Croatian practitioners reported encountering adverse effects related to antibiotic use in their patients, compared to 31.3% in Bosnia and 33.6% in Serbia. Amoxicillin with clavulanic acid was selected as a first-line antibiotic for medically health patients with no penicillin allergy, with 91.4% of Croatian practitioners reporting its use, compared to 84.7% of Bosnian and 79.5% of Serbian practitioners. Clindamycin was commonly utilized as an alternative for patients with penicillin allergies, being prescribed by 88.7% of Croatian dentists and 67.8% of Serbian dentists. Additionally, 56.3% of Bosnian dentists reported prescribing erythromycin in such cases. No statistically significant differences were observed in antibiotic knowledge among respondents based on their self-assessment of knowledge, the number of antibiotics prescribed weekly, the average duration of antibiotic therapy, or the experience of side effects (*p* > 0.05).

Table 3 presents an assessment of awareness, education, and prescribing practices related to antibiotics in dental medicine. Over 90% of respondents stated that they are adherent to current guidelines for antibiotic prescribing, are familiar with recommended dosages, and are aware of antimicrobial resistance. However, only 66.1% of dentists in Croatia felt adequately educated during their studies, compared to just 48.4% in Serbia and 46.6% in Bosnia. Notably, only 9.7% of dentists in Bosnia and Herzegovina prescribe antibiotics at patients’ request, while 22.3% of Croatian dentists and 25.4% of Serbian dentists do so.

Table 4 presents the prescribing patterns of antibiotics for certain oral conditions among respondents. The great majority (over 90%) prescribe antibiotics to patients with severe dentofacial abscesses, acute periapical infections, necrotizing ulcerative gingivitis, and periodontal abscesses with systemic signs of infection. Conversely, only 61.0% of them prescribe antibiotics to patients presenting with pericoronitis, and only 38.5% prescribe them during implant surgery involving bone augmentation.

Table 5 illustrates the prophylactic antibiotic prescribing practices among respondents. The majority of dental practitioners (over 94%) prescribe antibiotics before dental procedures for patients with a history of recurrent infective endocarditis or those with prosthetic heart valves or materials. On the other hand, less than 40% of respondents prescribe medication for patients with conditions such as artificial joints, chemotherapy or radiotherapy, HIV, and other immunodeficiencies, as shown in Table 5.

Table 6 shows the distribution of correctly answered questions regarding the use of antibiotics in dentistry. The knowledge assessment on the use of antibiotics in dentistry was structured with the answer options “Yes”, “No” and “I don’t know”. Respondents received one point for each correct answer (“Yes”), and zero points for incorrect answers (“No” and “I don’t know”) and their total score was then calculated.

Participants gave the most correct answers to the question on antibiotics as an adjunct in treating infections with fever, systemic spread, and lymph node involvement, with 90.5% of Croatian, 83.0% of Bosnian, and 90.8% of Serbian respondents answering correctly. Similarly, for the question on antibiotics alongside incision, drainage, and cause removal for rapidly spreading dentoalveolar infections, 88.7% of Croatian, 90.3% of Bosnian, and 96.5% of Serbian respondents answered correctly. Conversely, the lowest number of correct answers was given to the question stating that antimicrobial agents are not recommended for chronic dentoalveolar infections. The correct response rates were 69.9% for Croatian, 63.1% for Bosnian, and 66.8% for Serbian participants.

**Figure 1:**
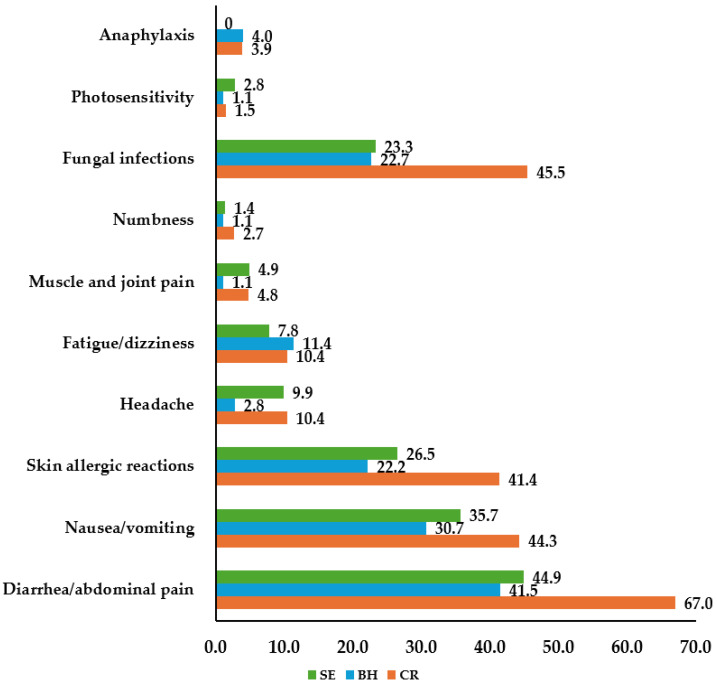
Reported side effects among patients associated with antibiotic usage.

Figure 1 illustrates the reported side effects associated with antibiotic use among patients. The most frequently reported side effect was diarrhea/abdominal pain, cited by 67.0% of Croatian, 41.5% of Bosnian, and 44.9% of Serbian dental practitioners.

**Figure 2:**
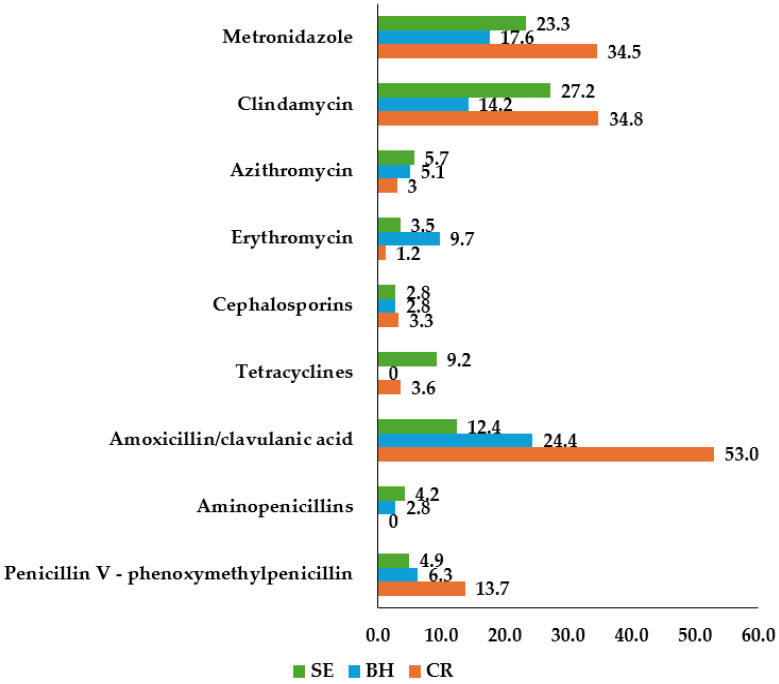
Antibiotics associated with reported side effects.

Figure 2 shows which antibiotics were most frequently associated with previously reported side effects from Figure 1. More than half of Croatian dentists (53.0%) reported amoxicillin with clavulanic acid as the cause, along with 24.4% of Bosnian and 12.4% of Serbian dentists. Other reported antibiotics included clindamycin (34.8% of Croatian, 14.2% of Bosnian, and 27.2% of Serbian dentists) and metronidazole (34.5% of Croatian, 17.6% of Bosnian, and 23.3% of Serbian dentists).

## 3. Discussion

The proper use of antibiotics in dental practice is essential for effectively treating odontogenic infections while minimizing the development of bacterial resistance. Achieving this requires prescribing antibiotics only when necessary and ensuring they are administered at the appropriate dosage [5]. This study aimed to compare and evaluate antibiotic prescription practices and knowledge among dental practitioners from Croatia, Bosnia and Herzegovina, and Serbia. Although previous studies have explored antibiotic prescription practices in these regions, none have simultaneously encompassed or systematically compared all three countries. Research has typically focused on individual nations or specific groups of dental professionals, leaving a gap in understanding how these regions differ in their knowledge and antibiotic prescribing behaviors. Consequently, there remains limited comprehensive analysis that contrasts these nations directly within a unified framework [24,25,26,27,28,29,30,31,32,33,34].

Dental practitioners from this study demonstrated competent antibiotic knowledge, with a total knowledge score of 6.40 ± 1.40 of a possible maximum score of 8. There were no statistically significant differences in knowledge among dental practitioners based on their country of practice. The knowledge test featured questions focusing on the use of antibiotics in clinical scenarios. One question addressed the recommendation of antibiotics as an adjunct in the definitive treatment of infections, particularly in cases with fever, systemic spread, and local lymph node involvement. Another question examined the recommendation of antibiotics in conjunction with incision, drainage, and removal of the cause for severe dentoalveolar infections that spread rapidly [3,4,5]. These questions were successfully answered by more than 83% of participants, highlighting a strong understanding of antibiotic use in these critical situations. On the other hand, the use of antibiotics is not justified in the treatment of chronic infections. Case reports and literature reviews indicate that removing the cause of the infection typically resolves the issue, allowing extraoral cutaneous sinus tracts to heal spontaneously [3,4,5]. This was recognized by only 69.9% of Croatian dentists, 63.1% of Bosnian dentists, and 66.8% of Serbian dentists, revealing a notable knowledge gap about antibiotic use for chronic infections among dental practitioners.

In this study, over 90% of respondents reported favorable practices regarding antibiotic use, including adherence to established guidelines for prescriptions, familiarity with recommended dosages, and awareness of antimicrobial resistance. A study conducted in Primorsko-Goranska County, Croatia, reported that 99.4% of dental practitioners demonstrated high awareness of antimicrobial resistance [27]. However, while 83.5% of dentists in a study involving Croatian and Bosnian practitioners indicated they utilized antibiotic guidelines, only 7.8% were able to accurately articulate valid guidelines [34]. Some studies have identified that a primary factor influencing dentists to prescribe antibiotics is the perceived pressure from patients to obtain antibiotic prescriptions [35,36]. In this study, 25.4% of Croatian, 9.7% of Bosnian, and 22.3% of Serbian dental practitioners reported prescribing antibiotics at the request of patients. Notably, an even greater proportion (43.4%) in a study conducted in Primorsko-Goranska County acknowledged similar practices [27]. These findings underscore a concerning trend in antibiotic prescribing practices influenced by patient requests across the examined regions.

Antibiotics are indicated in scenarios where an abscess rapidly disseminates beyond the dento-alveolar region into adjacent tissues, presenting with systemic signs and symptoms. Such cases often necessitate advanced management due to the risk of severe complications, including Ludwig’s angina and cellulitis [5]. Almost all participants (over 97.0%) in this study recognized this scenario and reported applying this understanding in their clinical practice. However, other studies in this region have shown varied results. Dental practitioners in a study from Primorsko-Goranska County prescribed antibiotics for periapical abscesses (84.7%) and periodontal abscesses (72.6%) [27]. A separate study on antibiotic prescriptions in emergency dental services in Zagreb, Croatia, indicated that 70.7% of antibiotic prescriptions were made for periapical periodontitis, acute apical abscess, or pulpitis [26]. Notably, one-quarter of participants in that study incorrectly prescribed antibiotics for pulpitis, which is not an appropriate indication. Additionally, another study among dental practitioners in Zagreb found that 44.05% of participants prescribed antibiotics for periapical or periodontal abscesses [30]. A 5-year national study among Croatian dental practices revealed that 22.0% of antibiotics were prescribed for inconclusive indications, while 29.79% were not prescribed following contemporary guidelines for the proper use of antibiotics [25].

Besides the therapeutic use of antibiotics, they are also recommended prophylactically before invasive dental procedures for certain groups of patients. The American Heart Association recommends antimicrobial prophylaxis before specific dental procedures for patients at increased risk of infective endocarditis if they have a prosthetic valve or implanted cardiac material, a history of previous infective endocarditis, certain congenital heart conditions, or valvopathy following a cardiac transplant [37]. In this study, most participants (over 94%) recognized previous or recurrent infective endocarditis and the presence of a prosthetic heart valve or material as correct indications for antibiotic prophylaxis. Various conditions, such as leukemia, HIV, chronic diseases like end-stage renal disease, patients on dialysis, those with uncontrolled diabetes, individuals receiving chemotherapy, radiation, steroids, immunosuppressive post-transplant medications, and those with genetic defects, can compromise immune function, making patients more susceptible to opportunistic infections. The prompt and effective management of dental infections in these patients is essential and may require collaboration with their healthcare specialists, with antibiotic prescription potentially justified in such cases [5]. However, current evidence does not indicate an increased risk of infection related to dental procedures or surgical site infections in this population [38,39,40,41]. Despite these uncertainties, a significant proportion of dentists in this study indicated these medical conditions as indications for the prophylactic use of medications. In this study, 49.4% of Croatian, 34.7% of Bosnian, and 17.0% of Serbian dental practitioners would prescribe antibiotic prophylaxis for patients with artificial joints. A systematic review of nine studies, along with additional literature, examined the association between dental procedures and the risk of artificial joint infections. The findings revealed no evidence supporting the use of antibiotic prophylaxis in reducing joint infection rates [42]. However, in patients with a history of complications from joint replacement surgeries undergoing dental procedures involving gingival manipulation or mucosal incision, prophylactic antibiotics should be considered only after consulting both the patient and the orthopedic surgeon. When antibiotics are necessary, the orthopedic surgeon should recommend the appropriate regimen and, if needed, write the prescription. Practitioners and patients should weigh the medical risks of performing dental treatments without antibiotics against the potential risks of frequent or unnecessary antibiotic use. This approach should be integrated into clinical decision-making, balancing evidence-based guidelines with professional judgment and patient preferences [43].

Preferences for the first-choice antibiotic for medically healthy patients differed among respondents based on their country of practice. Amoxicillin with clavulanic acid was the dominant choice, preferred by 91.4% of Croatian practitioners, compared to 84.7% of Bosnian and 79.5% of Serbian practitioners. These findings align with studies examining antibiotic prescriptions among Croatian dentists, indicating a strong preference for penicillin combined with clavulanic acid within this group [25,26]. This presents a significant issue, as Croatian dentists favor the use of the broad-spectrum co-amoxiclav, despite existing guidelines in dentistry recommending amoxicillin as the first-choice antibiotic [24]. This contrasts with Serbian dentists, who have been shown in previous studies to favor amoxicillin, while in our study only 15.2% selected amoxicillin as a first-line antibiotic [32].

Recent guidelines indicate that the second-choice antimicrobials include either metronidazole or a macrolide, such as clarithromycin, which provide better pharmacokinetics and tolerability compared to erythromycin. Clindamycin is effective against oral anaerobes but is associated with a higher incidence of gastrointestinal side effects, including diarrhea, and has been linked to an increased risk of *Clostridium difficile* infections. For some patients, clindamycin may be the only suitable antimicrobial option due to allergies or drug interactions. While cephalosporins can be used for oral infections, they do not offer any advantages over penicillin in treating dental infections and are less effective against anaerobic bacteria [6]. In this study, clindamycin was identified as the second-choice antibiotic for patients with penicillin allergies by 88.7% of Croatian dentists, whereas only 39.8% of Bosnian dentists and 67.8% of Serbian dentists preferred it. In this study, dental professionals from Bosnia and Herzegovina identified erythromycin (56.3%) and azithromycin (20.5%) as alternative antibiotic options. This contrasts with another study among Bosnian dental practitioners, who reported clindamycin (33.9%) as the most preferred alternative, followed by lincomycin (29.4%), erythromycin (17.2%), metronidazole (10.0%), and azithromycin (9.4%) [33]. In this study, Serbian dental professionals showed a preference for clindamycin (67.8%), erythromycin (45.2%), and azithromycin (20.5%). In contrast, another study conducted among Serbian dentists found that 61.4% used clindamycin, followed by azithromycin and erythromycin in 14.6% of cases [32]. Despite these practices among dental professionals, British guidelines clearly state that the routine prescribing of clindamycin, cephalosporins, or co-amoxiclav for dental infections is not recommended and should only occur under the direction of a specialist in oral/medical microbiology or infectious diseases [6].

In this study, 50.9% of Croatian dental practitioners reported observing antibiotic-related side effects in their patients, compared to 31.3% of Bosnian and 33.6% of Serbian practitioners. The most commonly reported side effect was diarrhea or abdominal pain, affecting 67.0% of Croatian, 41.5% of Bosnian, and 44.9% of Serbian dental practitioners. Notably, over half of Croatian practitioners (53.0%) identified amoxicillin with clavulanic acid as the primary cause of these side effects, while 24.4% of Bosnian and 12.4% of Serbian respondents attributed them to the same antibiotic. These findings highlight that gastrointestinal side effects, especially diarrhea and abdominal pain, are frequently associated with clavulanic acid use [44]. This underscores the importance of awareness among dental practitioners regarding the potential adverse effects of antibiotics and emphasizes the need for careful patient monitoring and management.

This study has several limitations that should be considered. First, the data were obtained through an online cross-sectional survey that relies on participants’ self-reporting of their knowledge and prescribing practices, which may introduce bias. There is also a risk of non-response bias, as people with less knowledge about the topic may have chosen not to participate, which could limit the representativeness of the results. Another limitation is the relatively small sample size with an uneven distribution of respondents from each country—most came from Croatia, while Bosnia and Herzegovina had the fewest participants. There was also a gender imbalance, with more women than men taking part in the survey. Another major limitation is the respondents’ varying knowledge of antibiotics and their use in dentistry, which is based on the different clinical guidelines of their respective institutions. This variability could contribute to discrepancies in responses and affect the generalizability of the study. These limitations suggest that the results should be interpreted with caution, and future research should seek a larger and more balanced sample from each country to ensure broader generalizability and mitigate potential bias. Another limitation of the study is that the ‘correct’ answers for all questions should be “yes”. This may lead to an overestimation of respondents’ knowledge and compliance and highlights the need for a more thorough assessment of actual clinical practice.

The study shows that although dental professionals in the region generally have a good understanding of the use of antibiotics, there are significant discrepancies between their theoretical knowledge and their actual prescribing practice. In addition, responses to certain questions may vary depending on the guidelines with which respondents are familiar, contributing to these discrepancies. These differences highlight the need for targeted education programs and awareness campaigns to bridge the gap between knowledge and behavior and ensure that physicians follow established guidelines. Such initiatives should focus on both the therapeutic and prophylactic use of antibiotics, dispel common misconceptions, and promote evidence-based decision-making. In addition, there is a clear need to develop comprehensive national guidelines for the use of antibiotics in dentistry in each country. These guidelines would serve to standardize practices, minimize inappropriate prescribing, and reduce the risk of antibiotic resistance, ultimately leading to safer and more effective patient care.

## 4. Materials and Methods

### 4.1. Study Design and Population

This cross-sectional electronic survey was conducted from 1 June to 30 August 2024, at the Department of Restorative Dentistry and Endodontics, School of Medicine, University of Split, Croatia. The data were collected using the Google survey tool (Google Forms, Google, Mountain View, CA, USA). The authors contacted respondents from Croatia, Bosnia and Herzegovina, and Serbia via available online email addresses from a Facebook group “Zubarolozi” (N = 12,900 dentists), invited them to participate in the study, and provided a link to the survey. Participation in the study was voluntary and anonymous, and a consent form was provided at the beginning. After the initial invitation, reminder emails were sent at two-week intervals. Participants were selected using a random sample, supplemented by a snowball method in which respondents were asked to forward the survey to interested colleagues.

The minimum necessary sample size (n = 375) was determined utilizing the Sample Size Calculator (RaoSoft^®^, Inc., Seattle, WA, USA), an online tool [45]. This calculation was based on an estimated population of dentists working in the Croatian, Serbian, and Bosnian Herzegovinian health systems (NCR = 3982, NBH = 575, NSE = 9412), an expected response rate of 50%, a confidence level of 95%, and a margin of error of 5% [46,47,48].

The study’s inclusion criteria encompassed dental practitioners from Croatia, Bosnia and Herzegovina, and Serbia who were willing and able to complete an online survey and had at least one year of clinical experience. Exclusion criteria included incomplete questionnaires, retired or non-practicing dental practitioners, and recently graduated dental students.

The study received ethical approval from the appropriate institutional bodies, adhering to all relevant guidelines and regulations, including the World Medical Association’s Declaration of Helsinki. The research protocol was reviewed and approved by the Ethics Committee of the University of Split School of Medicine, Croatia (Reference: Class: 029-01/24-02/0001, No.: 2181-198-03-04-24-0065) and approved on 6 June 2024. In writing the scientific paper, the authors followed the CHERRIES checklist and STROBE guidelines [49,50]. Informed consent was obtained from all participants via an online consent form embedded in the survey, which also indicated that the survey would take approximately 15 min to complete and included the number of questions. Participants were assured that their data would be treated confidentially by the researchers and used solely to publish the study.

### 4.2. Questionnaire

The questionnaire was developed and customized using insights from previous research on antibiotic prescribing practices in dental care, as well as contemporary guidelines on antibiotic use in dentistry [6,8,12,16,17,19,20,21,22,35,36]. To ensure content validity, the survey was thoroughly evaluated by a panel of experts, consisting of two university professors specializing in endodontics. After this review, a pilot study was conducted with a group of 30 dental practitioners to assess the clarity and comprehensibility of the questions. The pilot study confirmed that the questionnaire was easy to understand, and no further modifications were necessary. The pilot also determined that completing the survey took approximately 15 min. Notably, the participants in the pilot study were not included in the main research sample.

The questionnaire consisted of 70 questions divided into eight sections (Supplementary File S1). The first part comprised ten questions (Q1–10) that assessed the demographic and professional characteristics of the participants, as well as the country in which they practice. The second part with eight questions (Q11–18) presents an overview of key findings related to self-reported knowledge and prescribing practices concerning antibiotics in dental medicine, along with the most frequently prescribed antibiotics in clinical practice. In the third part of the questionnaire, participants answered 11 questions (Q19–29) to assess their awareness, training, and prescribing practices regarding antibiotics in dentistry. Possible response options included “Yes” or “No”. In the fourth section, respondents were asked 18 questions (Q30–47) about antibiotic prescribing patterns for curative purposes in medically healthy patients. In the fifth section, 13 scenarios on patients with various conditions were presented (Q48–60), asking respondents whether they would prescribe antibiotics for prophylactic purposes. In both sections, respondents were offered the options “Yes” and “No”. In the sixth section, 8 questions with the answer options “Yes”, “No”, and “I don’t know” were asked to determine knowledge about the use of antibiotics in dentistry (Q61–68). Respondents were assigned one point for each correct answer. At the end, the total knowledge score of the participants was calculated. According to Bloom’s taxonomy of knowledge, we categorized those who scored between 80 and 100% (6.4 to 8 points) as having “good knowledge”, while those who scored between 60 and 79% (6.3 to 4.8 points) were labeled as having “moderate knowledge”. Conversely, respondents who scored less than 60% (0 to 4.7 points) were labeled as having “poor knowledge” [51]. In the seventh section, respondents answered one question (Q69) about the reported side effects of antibiotic use in their patients, while the final section (Q70) identified the antibiotics responsible for these effects in their patients.

### 4.3. Data Analysis

Data analysis was performed using SPSS Statistics version 26.0 (IBM Corp., Armonk, NY, USA), with statistical significance set at a *p*-value of less than 0.05. The normality of the data was checked using the Kolmogorov–Smirnov test. Descriptive statistics were used to determine the basic statistical parameters, including mean, standard deviation, median, minimum, and maximum values. The Mann–Whitney test was used to compare the knowledge values between two groups, while the Kruskal–Wallis test with the corresponding post hoc analysis was used to compare the mean values between three or more groups. The differences between respondents in terms of socio-demographic and occupational characteristics in the different countries were analyzed using the chi-square test.

## 5. Conclusions

This study revealed that factors such as female gender, specialization in pediatric dentistry, and practice settings in urban areas were significantly associated with enhanced antibiotic knowledge among dental practitioners. In contrast, other variables, including country of practice, education level, years of experience, self-assessed knowledge, frequency of antibiotic prescriptions, the average duration of antibiotic therapy, and experiences with side effects, did not exhibit a statistically significant effect on antibiotic knowledge. Although nearly all respondents stated that they follow the guidelines, the results of this study indicate that this is not consistently reflected in their practice. These results emphasize the necessity of developing clear guidelines for antibiotic use, particularly within primary care settings, across all countries. Furthermore, implementing targeted educational initiatives to bolster antibiotic awareness among specific demographic and professional groups within the dental community is crucial for advancing overall antibiotic stewardship and optimizing patient care.

## Figures and Tables

**Table 1:** Demographic and professional characteristics of respondents by country and antibiotic knowledge scores.

Characteristics		Total (N = 795)	Antibiotic Knowledge	*p*-Value ^a^	CR (N = 336)	BH (N = 176)	SE (N = 283)	*p*-Value ^b^
Sex	Man	226 (28.4)	6.08 (1.45)	≤0.001 *	98 (29.2)	55 (31.3)	73 (25.8)	0.418
	Woman	569 (71.6)	6.55 (1.41)		238 (70.8)	121 (68.8)	210 (74.2)	
Age group (years)	≤30	230 (28.9)	6.55 (1.30)	0.201	121 (36.0)	31 (17.6)	78 (27.6)	≤0.001 *
	31–40	292 (36.7)	6.18 (1.51)		92 (27.4)	90 (51.1)	110 (38.9)	
	41–50	187 (23.5)	6.56 (1.41)		73 (21.7)	39 (22.2)	75 (26.5)	
	≥51	86 (10.8)	6.51 (1.52)		50 (14.9)	16 (9.1)	20 (7.1)	
Academic qualification	DMD	648 (81.5)	6.40 (1.47)	0.480	272 (81.0)	131 (74.4)	245 (86.6)	0.005 *
	MSc/PhD	147 (18.5)	6.49 (1.51)		64 (19.0)	45 (25.6)	38 (13.4)	
Dental specialty	General	600 (75.4)	6.37 (1.43) ^a^	0.002 *	260 (77.4)	129 (73.4)	211 (74.6)	0.013 *
	Endodontics	34 (4.3)	6.97 (1.16) ^g^		15 (4.5)	7 (4.0)	12 (4.2)	
	Oral surgery	53 (60.6)	6.52 (1.42) ^b,h^		19 (5.7)	14 (7.9)	20 (7.1)	
	Oral medicine	3 (0.4)	5.67 (1.52)c		3 (0.9)	0 (0)	0 (0)	
	Pediatrics	26 (3.3)	7.42 (0.75) ^a–f^		5 (1.5)	7 (4.0)	14 (4.9)	
	Orthodontics	23 (2.9)	6.22 (1.73) ^d^		7 (2.1)	8 (4.5)	8 (2.8)	
	Periodontics	17 (2.1)	6.53 (1.28) ^d^		7 (2.1)	2 (1.1)	8 (2.8)	
	Prosthodontics	33 (4.2)	6.0 (1.34) ^e,g,h^		14 (4.2)	9 (5.1)	10 (3.5)	
	Family Dentistry	6 (0.8)	5.76 (2.65) ^f^		6 (1.8)	0 (0)	0 (0)	
Practice setting	Private practice	451 (56.7)	6.38 (1.47)	0.188	116 (34.5)	135 (76.7)	200 (70.7)	≤0.001 *
	Health center	273 (34.3)	6.40 (1.39)		169 (50.3)	27 (15.3)	77 (27.2)	
	Secondary/tertiary care	71 (8.9)	6.71 (1.40)		51 (15.2)	14 (8.0)	6 (2.1)	
Area of working	Urban	649 (81.6)	6.47 (1.43)	0.034 *	266 (79.2)	138 (78.4)	245 (86.6)	0.027 *
	Rural	146 (18.4)	6.18 (1.46)		70 (20.8)	38 (21.6)	38 (13.4)	
Clinical working experience	≤10 years	469 (57.9)	6.44 (1.34)	0.597	185 (55.1)	90 (51.1)	185 (65.3)	0.004 *
	>10 years	335 (42.1)	6.38 (1.56)		151 (44.9)	86 (48.9)	98 (34.7)	
Working hours with patients per day	≤6 h	275 (34.6)	6.34 (1.50)	1.788	110 (32.7)	53 (30.1)	112 (39.6)	0.075
	>6 h	520 (65.4)	6.40 (1.40)		226 (67.3)	123 (69.9)	171 (60.4)	
Number of patients per day	≤10 patients	275 (47.2)	6.35 (1.50)	0.215	138 (41.1)	79 (44.9)	158 (55.8)	≤0.001 *
	>10 patients	420 (52.8)	6.47 (2.38)		198 (58.9)	97 (55.1)	25 (44.2)	

Data are presented as numbers (percentages) or mean (SD). Statistical significance was determined using the Mann–Whitney test ^a^ or Kruskal–Wallis one-way ANOVA ^a^ for the knowledge values and the chi-square test ^b^ for the differences between the countries in the demographic and occupational factors. Groups with statistically significant differences in post hoc tests are labeled with the same superscript lowercase letter. Abbreviations: CR—Croatia; BH—Bosnia and Herzegovina; SE—Serbia. * *p* < 0.05 indicates statistical significance.

**Table 2:** Self-reported knowledge and prescribing practices related to antibiotics in dental medicine.

Characteristic		Total (N = 795)	CR (N = 336)	BH (N = 176)	SE (N = 283)
Self-assessment of personal knowledge and rational prescribing patterns regarding antibiotics	Poor	15 (1.9)	4 (1.2)	1 (0.6)	10 (3.5)
	Moderate	566 (71.2)	222 (66.1)	124 (70.5)	220 (77.7)
	Good	214 (26.9)	110 (32.7)	51 (29.0)	53 (18.7)
Source of antibiotic information	Dental school	611 (76.9)	275 (81.8)	129 (73.3)	207 (73.1)
	Seminars and congresses	315 (39.6)	127 (37.8)	74 (42.1)	114 (40.3)
	Colleagues	299 (37.6)	106 (31.5)	82 (46.6)	111 (39.2)
	Pharmacists	147 (18.5)	49 (14.6)	51 (28.9)	47 (16.7)
	Internet	249 (31.3)	97 (28.9)	77 (43.8)	81 (28.6)
	Articles and scientific papers	347 (43.6)	143 (42.6)	94 (53.4)	110 (38.9)
Average number of prescribed antibiotics per week	0–1	396 (49.8)	156 (46.4)	77 (43.8)	163 (57.6)
	2–5	365 (45.9)	165 (49.1)	86 (48.9)	114 (40.3)
	>5	34 (4.3)	15 (4.5)	13 (7.4)	6 (2.1)
The average duration of prescribed antibiotic therapy for dentoalveolar infection	≤ 5 days	172 (21.6)	27 (8.0)	47 (26.7)	98 (34.6)
	5–7 days	533 (67.1)	253 (75.3)	106 (60.2)	174 (61.5)
	≥ 7 days	90 (11.3)	56 (16.7)	23 (13.1)	11 (3.9)
The most common procedures that require an antibiotic prescription *	Pediatric Dentistry	129 (16.2)	37 (11.1)	20 (11.4)	72 (25.4)
	Endodontics and Restorative Dentistry	453 (57.0)	244 (72.6)	87 (49.4)	122 (43.1)
	Oral surgery	666 (83.8)	257 (76.5)	163 (92.6)	246 (86.9)
	Oral medicine	37 (4.7)	5 (1.4)	19 (10.8)	13 (4.6)
	Orthodontics	3 (0.4)	1 (0.1)	0 (0)	2 (0.1)
	Periodontology	219 (7.5)	102 (30.4)	32 (18.2)	85 (30.1)
Patient feedback regarding antibiotic side effects	Yes	321 (40.4)	171 (50.9)	55 (31.3)	95 (33.6)
	No	474 (59.6)	165 (49.1)	121 (68.8)	188 (66.4)
A first-choice antibiotic for treating dentoalveolar infections in healthy patients with no allergy to penicillin *	Amoxicillin/clavulanic acid	681 (85.7)	307 (91.4)	149 (84.7)	225 (79.5)
	Clindamycin	130 (16.4)	30 (8.9)	28 (15.9)	72 (25.4)
	Metronidazole	182 (22.9)	58 (17.3)	53 (30.1)	71 (25.1)
	Aminopenicillin	90 (11.3)	30 (8.9)	17 (9.7)	43 (15.2)
	Penicillin V	95 (11.5)	35 (10.4)	19 (10.8)	41 (14.5)
	Other	182 (22.9)	58 (17.3)	53 (30.1)	71 (25.1)
A first-choice antibiotic for treating dentoalveolar infections in healthy patients with an allergy to penicillin *	Cephalosporin	73 (9.2)	40 (11.9)	17 (9.6)	16 (5.6)
	Erythromycin	245 (30.8)	18 (5.4)	99 (56.3)	128 (45.2)
	Clarithromycin	44 (5.5)	13 (3.9)	16 (9.1)	15 (5.3)
	Azithromycin	122 (15.3)	28 (8.3)	36 (20.5)	58 (20.5)
	Clindamycin	560 (70.4)	298 (88.7)	70 (39.8)	192 (67.8)
	Metronidazole	117 (14.7)	38 (11.3)	22 (12.5)	57 (20.1)
	Tetracycline	21 (2.6)	3 (0.9)	0 (0)	18 (6.4)

Data are presented as numbers (percentages). * Multiple answers possible. Abbreviations: CR—Croatia; BH—Bosnia and Herzegovina; SE—Serbia.

**Table 3:** Assessment of awareness, education, and prescribing practices related to antibiotics in dental medicine.

Characteristic	Total (N = 795)	CR (N = 336)	BH (N = 176)	SE (N = 283)
Adherence to the current guidelines on proper curative and prophylactic prescribing of antibiotics in dental medicine (“Yes”)	759 (95.5)	324 (96.4)	167 (94.9)	268 (94.7)
Following updates to the guidelines on the proper use of antibiotics in dental medicine (“Yes”)	569 (71.6)	244 (72.6)	105 (59.7)	220 (77.7)
Sufficiently educated on the topic of antibiotics and their application in dental medicine during both undergraduate and postgraduate studies (“Yes”)	441 (55.5)	222 (66.1)	82 (46.6)	137 (48.4)
Interested in future education on the topic of antibiotics and their application in dental medicine (“Yes”)	755 (95.0)	314 (93.5)	165 (93.8)	276 (97.5)
Awareness of antimicrobial resistance (“Yes”)	758 (95.3)	328 (97.6)	170 (96.6)	260 (91.9)
Taking antimicrobial resistance into consideration when prescribing antibiotic therapy (“Yes”)	684 (86.0)	294 (87.5)	159 (90.3)	231 (81.6)
Considering antimicrobial resistance a serious threat to public health (“Yes”)	780 (98.1)	323 (96.1)	176 (100.0)	281 (99.3)
Prescribing antibiotics at the request of patients (“Yes”)	164 (20.6)	75 (22.3)	17 (9.7)	72 (25.4)
Prescribing antibiotics when not indicated (“Yes”)	125 (15.7)	53 (15.8)	32 (18.2)	40 (14.1)
Considering that antibiotics are overly and indiscriminately prescribed in dental medicine (“Yes”)	699 (87.9)	301 (89.6)	168 (95.5)	230 (81.3)
Familiarity with the recommended doses of various types of antibiotics (“Yes”)	741 (93.2)	314 (93.5)	163 (92.6)	264 (93.3)

Data are presented as numbers (percentages). Abbreviations: CR—Croatia; BH—Bosnia and Herzegovina; SE—Serbia.

**Table 4:** Prescribing patterns of antibiotics for curative purposes in medically healthy patients.

Condition	Total (N = 795)	CR (N = 336)	BH (N = 176)	SE (N = 283)
Severe dentofacial abscesses, rapidly spreading cellulitis, and Ludwig’s angina (“Yes”)	780 (98.1)	329 (97.9)	173 (98.3)	278 (98.2)
Acute periapical infections with systemic signs of inflammation, elevated temperature, and enlarged lymph nodes. (“Yes”)	783 (98.5)	328 (97.6)	175 (99.4)	280 (98.9)
Osteomyelitis (“Yes”)	675 (84.9)	285 (84.8)	156 (88.6)	234 (82.7)
Medication-related osteonecrosis of the jaw with secondary infection (“Yes”)	615 (77.4)	277 (82.4)	131 (74.4)	207 (73.1)
Pericoronitis (“Yes”)	485 (61.0)	208 (61.9)	90 (51.1)	187 (66.1)
Dry socket (localized osteitis) (“Yes”)	251 (31.6)	116 (34.5)	60 (34.1)	75 (26.5)
Acute sinusitis (“Yes”)	498 (62.6)	181 (53.9)	128 (72.7)	189 (66.8)
Acute bacterial sialadenitis (“Yes”)	629 (79.1)	269 (80.1)	134 (76.1)	226 (77.9)
Gingivitis (“Yes”)	61 (7.7)	16 (4.8)	13 (7.4)	32 (11.3)
Necrotizing ulcerative gingivitis with systemic signs of inflammation, elevated temperature, and enlarged lymph nodes (“Yes”)	747 (94.0)	320 (95.2)	159 (90.3)	268 (94.7)
Periodontal abscesses with systemic signs of inflammation, elevated temperature, and enlarged lymph nodes (“Yes”)	769 (96.7)	331 (98.5)	164 (93.2)	274 (96.8)
Peri-implant mucositis (“Yes”)	229 (28.8)	90 (26.8)	56 (31.8)	83 (99.3)
Peri-implantitis (“Yes”)	418 (52.6)	185 (55.1)	87 (49.4)	146 (51.6)
Implant placement (“Yes”)	350 (44.0)	129 (38.4)	115 (65.3)	106 (37.5)
Intraoral bone augmentation before implant placement (“Yes”)	306 (38.5)	125 (37.2)	91 (51.7)	90 (31.8)
Acute pulpitis (“Yes”)	28 (3.5)	17 (5.1)	5 (2.8)	6 (2.1)
Tooth avulsion (“Yes”)	403 (50.7)	169 (50.3)	105 (59.7)	129 (45.6)
Oroantral fistula (“Yes”)	379 (47.7)	163 (48.5)	92 (52.3)	124 (43.8)

Data are presented as numbers (percentages). Abbreviations: CR—Croatia; BH—Bosnia and Herzegovina; SE—Serbia.

**Table 5:** Prophylactic antibiotic prescribing practices for interventional dental procedures in medically compromised patients.

Condition	Total (N = 795)	CR (N = 336)	BH (N = 176)	SE (N = 283)
Previous or recurrent infective endocarditis (“Yes”)	781 (98.2)	329 (97.9)	176 (100.0)	276 (97.5)
Prosthetic heart valve or material (“Yes”)	754 (94.8)	319 (94.9)	168 (95.5)	267 (94.3)
Heart transplant recipients who develop heart valve disease (“Yes”)	718 (91.3)	321 (95.5)	143 (81.3)	254 (89.8)
Cardiac pacemakers, penile implants, breast implants, or intraocular implants (“Yes”)	155 (19.5)	59 (17.6)	45 (25.6)	51 (18.0)
Chemotherapy (“Yes”)	317 (39.9)	156 (46.4)	54 (30.7)	107 (37.8)
Radiotherapy (“Yes”)	286 (36.0)	134 (42.6)	46 (26.1)	97 (34.3)
Transplanted organs (“Yes”)	508 (63.9)	250 (74.4)	102 (58.0)	156 (55.1)
HIV (“Yes”)	205 (25.8)	75 (22.3)	51 (29.0)	79 (27.9)
Diabetes (“Yes”)	125 (15.7)	43 (12.8)	32 (18.2)	50 (17.7)
Renal dialysis (“Yes”)	224 (28.2)	88 (26.2)	33 (18.8)	103 (36.4)
Artificial joint (“Yes”)	275 (34.6)	166 (49.4)	61 (34.7)	48 (17.0)
Devices for intravenous access (central venous lines/permanent catheters used for parenteral nutrition or chemotherapy, and hemodialysis catheters) (“Yes”)	250 (31.4)	109 (32.4)	56 (51.8)	85 (30.0)
Autoimmune disease (“Yes”)	111 (14.0)	46 (13.7)	18 (10.2)	47 (16.6)

Data are presented as numbers (percentages). Abbreviations: CR—Croatia; BH—Bosnia and Herzegovina; SE—Serbia.

**Table 6:** Assessment of knowledge on antibiotic use in dentistry: frequency distribution of correct responses by dental practitioners.

Question	Total (N = 795)	CR (N = 336)	BH (N = 176)	SE (N = 283)
Antibiotics are recommended as an adjunct in the definitive treatment of infections, especially when there is fever, evidence of systemic spread of infection, and local involvement of lymph nodes. (“Yes”) [6]	707 (88.9)	304 (90.5)	146 (83.0)	257 (90.8)
Antibiotics are recommended alongside incision, drainage, and removal of the cause for severe dentoalveolar infections that spread rapidly. (“Yes”) [6]	730 (91.8)	298 (88.7)	159 (90.3)	273 (96.5)
Routine prescription of clindamycin, cephalosporins, or co-amoxiclav for dental infections is not recommended and should only be carried out based on specialist guidance (“Yes”) [6]	562 (70.7)	228 (67.9)	114 (64.8)	220 (77.7)
Antimicrobial agents are not recommended for chronic dentoalveolar infections (“Yes”) [6]	535 (67.3)	235 (69.9)	111 (63.1)	189 (66.8)
Penicillin, such as phenoxymethylpenicillin or amoxicillin, is the first-choice antibiotic for dentoalveolar infections (“Yes”) [6]	723 (90.9)	291 (86.6)	163 (92.6)	269 (95.1)
The second-choice antimicrobial agent for dentoalveolar infections is either metronidazole or a macrolide, such as clarithromycin (“Yes”) [6]	591 (74.3)	241 (71.7)	143 (81.3)	207 (73.1)
For antibiotic prophylaxis, patients are given 2 g of oral amoxicillin 30–60 min before the procedure if the dental procedure involves manipulation of gingival tissue or the periapical region of the tooth or perforation of the oral mucosa (“Yes”) [8,9]	673 (84.7)	297 (88.4)	156 (88.6)	220 (77.7)
In case of penicillin allergy, alternative antibiotic prophylaxis includes cephalexin 2 g orally, azithromycin/clarithromycin 500 mg orally, or doxycycline 100 mg orally (“Yes”) [8,9]	578 (72.7)	222 (66.1)	137 (77.8)	219 (77.4)

Data are presented as numbers (percentages). Abbreviations: CR—Croatia; BH—Bosnia and Herzegovina; SE—Serbia.

## Data Availability

The data that support the findings of this study are available upon request from the corresponding author.

## Supplementary Materials

The following supporting information can be downloaded at: https://www.mdpi.com/article/10.3390/antibiotics13111061/s1, Supplementary File S1: Questionnaire in Croatian and English.

## References

[1] Oberoi S.S., Dhingra C., Sharma G., Sardana D. (2015). Antibiotics in Dental Practice: How Justified Are We?. Int. Dent. J..

[2] Lockhart P.B., Tampi M.P., Abt E., Aminoshariae A., Durkin M.J., Fouad A.F., Gopal P., Hatten B.W., Kennedy E., Lang M.S. (2019). Evidence-Based Clinical Practice Guideline on Antibiotic Use for the Urgent Management of Pulpal- and Periapical-Related Dental Pain and Intraoral Swelling: A Report from the American Dental Association. J. Am. Dent. Assoc..

[3] Ahmadi H., Ebrahimi A., Ahmadi F. (2021). Antibiotic Therapy in Dentistry. Int. J. Dent..

[4] Buonavoglia A., Leone P., Solimando A.G., Fasano R., Malerba E., Prete M., Corrente M., Prati C., Vacca A., Racanelli V. (2021). Antibiotics or No Antibiotics, That Is the Question: An Update on Efficient and Effective Use of Antibiotics in Dental Practice. Antibiotics.

[5] Contaldo M., D’Ambrosio F., Ferraro G.A., Di Stasio D., Di Palo M.P., Serpico R., Simeone M. (2023). Antibiotics in Dentistry: A Narrative Review of the Evidence beyond the Myth. Int. J. Environ. Res. Public Health.

[6] Palmer N. (2020). (Ed) Antimicrobial Prescribing in Dentistry: Good Practice Guidelines.

[7] Thornhill M., Prendergast B., Dayer M., Frisby A., Baddour L.M. (2024). Endocarditis Prevention: Time for a Review of NICE Guidance. Lancet Reg. Health Eur..

[8] American Academy of Pediatric Dentistry (2023). Antibiotic prophylaxis for dental patients at risk for infection. The Reference Manual of Pediatric Dentistry.

[9] Delgado V., Ajmone Marsan N., de Waha S., Bonaros N., Brida M., Burri H., Caselli S., Doenst T., Ederhy S., Erba P.A. (2023). ESC Scientific Document Group. 2023 ESC Guidelines for the Management of Endocarditis. Eur. Heart J..

[10] Thompson W., Tonkin-Crine S., Pavitt S.H., McEachan R.R.C., Douglas G.V.A., Aggarwal V.R., Sandoe J.A.T. (2019). Factors associated with antibiotic prescribing for adults with acute conditions: An umbrella review across primary care and a systematic review focusing on primary dental care. J. Antimicrob. Chemother..

[11] Martine C., Sutherland S., Born K., Thompson W., Teoh L., Singhal S. (2024). Dental Antimicrobial Stewardship: A Qualitative Study of Perspectives Among Canadian Dentistry Sector Leaders and Experts in Antimicrobial Stewardship. JAC Antimicrob Resist..

[12] Ramachandran P., Rachuri N.K., Martha S., Shakthivel R., Gundala A., Battu T.S. (2019). Implications of Overprescription of Antibiotics: A Cross-Sectional Study. J. Pharm. Bioallied Sci..

[13] Săndulescu O., Preoțescu L.L., Streinu-Cercel A., Şahin G.Ö., Săndulescu M. (2024). Antibiotic Prescribing in Dental Medicine—Best Practices for Successful Implementation. Trop. Med. Infect. Dis..

[14] Thompson W., Williams D., Pulcini C., Sanderson S., Calfon P., Verma M. (2020). The Essential Role of the Dental Team in Reducing Antibiotic Resistance.

[15] Hartshorne J. (2021). Antibiotic Stewardship in Dentistry—Review of Evidence-Based Clinical Recommendations on Appropriate Antibiotic Prescribing in Dental Practice. Part 1: The Antibiotic Resistance Crisis and the Principles and Practices of Appropriate Antibiotic Prescribing. Int. Dent. Afr. Ed..

[16] Bajalan A., Bui T., Salvadori G., Marques D., Schumacher A., Rösing C.K., Dahle U.R., Petersen F.C., Ricomini-Filho A.P., Nicolau B.F. (2022). Awareness Regarding Antimicrobial Resistance and Confidence to Prescribe Antibiotics in Dentistry: A Cross-Continental Student Survey. Antimicrob. Resist. Infect. Control.

[17] Al-Khatib A., AlMohammad R.A. (2022). Dentists’ Habits of Antibiotic Prescribing May Be Influenced by Patient Requests for Prescriptions. Int. J. Dent..

[18] Chehabeddine N., Lahoud N., Noujeim Z.E.F., Zeidan R.K., El Toum S., Maison P., Saleh N. (2022). An Evaluation of Prophylactic and Therapeutic Antibiotic Prescribing in Lebanese Dental Practice. Int. J. Pharm. Pract..

[19] Marah Z.A.A., Abdulkareem A.A., Gul S.S., Alshami M.L. (2022). A Survey of Systemic Antibiotic Prescription Patterns Amongst Iraqi Dentists. Int. Dent. J..

[20] Loume A., Gardelis P., Zekeridou A., Giannopoulou C. (2023). A Survey on Systemic Antibiotic Prescription among Dentists in Romandy. Swiss Dent. J..

[21] Rodríguez-Fernández A., Vázquez-Cancela O., Piñeiro-Lamas M., Herdeiro M.T., Figueiras A. (2023). Zapata-Cachafeiro, M. Magnitude and Determinants of Inappropriate Prescribing of Antibiotics in Dentistry: A Nationwide Study. Antimicrob. Resist. Infect. Control.

[22] Karobari M.I., Khijmatgar S., Bhandary R., Krishna Nayak U.S., Del Fabbro M., Horn R., Marya A. (2021). A Multicultural Demographic Study to Analyze Antibiotic Prescription Practices and the Need for Continuing Education in Dentistry. Biomed. Res. Int..

[23] Becker K., Gurzawska-Comis K., Klinge B., Lund B., Brunello G. (2024). Patterns of Antibiotic Prescription in Implant Dentistry and Antibiotic Resistance Awareness Among European Dentists: A Questionnaire-Based Study. Clin. Oral Implants Res..

[24] Šutej I., Bašić K., Šegović S., Peroš K. (2024). Antibiotic Prescribing Trends in Dentistry during Ten Years’ Period—Croatian National Study. Antibiotics.

[25] Petrac L., Gvozdanovic K., Perkovic V., Petek Zugaj N., Ljubicic N. (2024). Antibiotics Prescribing Pattern and Quality of Prescribing in Croatian Dental Practices—5-Year National Study. Antibiotics.

[26] Bjelovučić R., Par M., Rubčić D., Marović D., Prskalo K., Tarle Z. (2019). Antibiotic Prescription in Emergency Dental Service in Zagreb, Croatia: A Retrospective Cohort Study. Int. Dent. J..

[27] Farkaš M., Ivančić Jokić N., Mavrinac M., Tambić Andrašević A. (2021). Antibiotic Prescribing Habits and Antimicrobial Resistance Awareness of Dental Practitioners in Primorsko-Goranska County, Croatia. Microb. Drug Resist..

[28] Šimundić Munitić M., Šutej I., Ćaćić N., Tadin A., Balić M., Bago I., Poklepović Peričić T. (2021). Knowledge and Attitudes of Croatian Dentists Regarding Antibiotic Prescription in Endodontics: A Cross-Sectional Questionnaire-Based Study. Acta Stomatol. Croat..

[29] Sović J., Šegović S., Pavelić B., Bago I., Šutej I., Tomašić I. (2024). Patterns of Antibiotic Prescription in Endodontic Therapy in the Republic of Croatia. Antibiotics.

[30] Perić M., Perković I., Romić M., Simeon P., Matijević J., Mehičić G.P., Krmek S.J. (2015). The Pattern of Antibiotic Prescribing by Dental Practitioners in Zagreb, Croatia. Cent. Eur. J. Public Health.

[31] Roganović J., Barać M. (2024). Rational Antibiotic Prescribing Is Underpinned by Dental Ethics Principles: Survey on Postgraduate and Undergraduate Dental Students’ Perceptions. Antibiotics.

[32] Drobac M., Otasevic K., Ramic B., Cvjeticanin M., Stojanac I., Petrovic L. (2021). Antibiotic Prescribing Practices in Endodontic Infections: A Survey of Dentists in Serbia. Antibiotics.

[33] Galić M., Miletić I., Poklepović Peričić T., Rajić V., Većek Jurčević N.N., Pribisalić A. (2024). Medvedec Mikić, I. Antibiotic Prescribing Habits in Endodontics among Dentists in the Federation of Bosnia and Herzegovina—A Questionnaire-Based Study. Antibiotics.

[34] Ćorić A., Grgić S., Kostić S., Vukojević K., Zovko R., Radica N., Markotić F. (2020). Attitudes of Dental Practitioners towards Antimicrobial Therapy in Croatia and Bosnia and Herzegovina. Eur. J. Dent. Educ..

[35] Böhmer F., Hornung A., Burmeister U., Köchling A., Altiner A., Lang H., Löffler C. (2021). Factors, Perceptions and Beliefs Associated with Inappropriate Antibiotic Prescribing in German Primary Dental Care: A Qualitative Study. Antibiotics.

[36] Aly M.M., Elchaghaby M.A. (2021). The Prescription Pattern and Awareness about Antibiotic Prophylaxis and Resistance among a Group of Egyptian Pediatric and General Dentists: A Cross-Sectional Study. BMC Oral Health.

[37] Wilson W.R., Gewitz M., Lockhart P.B., Bolger A.F., DeSimone D.C., Kazi D.S., Couper D.J., Beaton A., Kilmartin C., Miro J.M. (2021). Prevention of Viridans Group Streptococcal Infective Endocarditis: A Scientific Statement from the American Heart Association. Circulation.

[38] Joint Formulary Committee (2019). British National Formulary.

[39] Barasch A., Safford M.M., Litaker M.S., Gilbert G.H. (2008). Risk factors for oral postoperative infection in patients with diabetes. Spec. Care Dent..

[40] Thorn J.J., Hansen H.S., Specht L., Bastholt L. (2000). Osteoradionecrosis of the jaws: Clinical characteristics and relation to the field of irradiation. J. Oral Maxillofac. Surg..

[41] Porter S.R., Scully C., Luker J. (1993). Complications of dental surgery in persons with HIV disease. Oral Surg. Oral Med. Oral Pathol..

[42] Rademacher W.M., Walenkamp G.H., Moojen D.J., Hendriks J.G., Goedendorp T.A., Rozema F.R. (2017). Antibiotic prophylaxis is not indicated prior to dental procedures for the prevention of periprosthetic joint infections. Acta Orthop..

[43] Sollecito T.P., Abt E., Lockhart P.B., Truelove E., Paumier T.M., Tracy S.L., Tampi M., Beltrán-Aguilar E.D., Frantsve-Hawley J. (2015). The use of prophylactic antibiotics prior to dental procedures in patients with prosthetic joints. J. Am. Dent. Assoc..

[44] Huttner A., Bielicki J., Clements M.N., Frimodt-Møller N., Muller A.E., Paccaud J.P., Mouton J.W. (2020). Oral amoxicillin and amoxicillin-clavulanic acid: Properties, indications and usage. Clin. Microbiol. Infect..

[45] Raosoft Sample Size Calculator. http://www.raosoft.com/samplesize.html.

[46] Croatian Health Statistics Yearbook. Croatian Institute of Public Health, 2021. https://www.hzjz.hr/wp-content/uploads/2023/05/HZSLj_-_2021_v._05.2023.pdf.

[47] Health Statistics Annual Federation of Bosnia and Herzegovina, 2022. https://www.zzjzfbih.ba/wp-content/uploads/2023/10/Godisnjak-2022_web.pdf.

[48] Serbian Dental Chamber. https://www.stomkoms.org/cr/articles/clanstvo/baza-podataka/.

[49] Eysenbach G. (2004). Improving the quality of Web surveys: The Checklist for Reporting Results of Internet E-Surveys (CHERRIES). J. Med. Internet Res..

[50] Cuschieri S. (2019). The STROBE guidelines. Saudi J. Anaesth..

[51] Bloom B.S. (1968). Learning for mastery. Instruction and curriculum. Regional education laboratory for the Carolinas and Virginia. Eval. Comment.

