# Supplementary material for: Antibiotics Knowledge and Prescription Patterns Among Dental Practitioners in Croatia, Bosnia and Herzegovina, and Serbia: A Comparative E-Survey with a Focus on Medically Healthy and Compromised Patients
Source: Antibiotics (Basel). 2024 Nov 8;13(11):1061. doi: 10.3390/antibiotics13111061 (PMC11591130; doi:10.3390/antibiotics13111061)
Supplement: Supplementary file 1 [file antibiotics-13-01061-s001.zip › antibiotics-3260703-supplementary.pdf]

antibiotics

MDPI

Supplementary Materials

Znanje i praksa propisivanja antibiotika među doktorima dentalne medicine u Hrvatskoj, Bosni i Hercegovini i Srbiji (upitnik)

1. Spol: a) M b) Ž
2. Dob (godine): ____
3. Stupanj obrazovanja:
   a) Dr. med. dent. b) Mr. sc./spec. mag. c) Dr. sc.
4. Specijalizacija:
   a) Bez specijalizacije b) Endodoncija c) Oralna kirurgija d) Protetika
   e) Oralna medicina f) Pedodoncija g) Parodontologija
   h) Ortodoncija i) Obiteljska
5. Radno mjesto:
   a) Privatna ordinacija b) Dom zdravlja c) Bolnica/poliklinika/fakultet
6. Godine radnog iskustva:
   a) 1-5 godina b) 6-10 godina c) 11-20 godina d) ≥21 godina
7. Broj sati rada s pacijentima u danu:
   a) ≤6 sati b) >6 sati
8. Broj pacijenata u danu:
   a) ≤10 pacijenata b) >10 pacijenata
9. Mjesto rada:
   a) Urbano područje b) Ruralno područje
10. Država u kojoj radite:
   a) Hrvatska b) Bosna I Hercegovins c) Srbija
11. Procijenite vlastito znanje i sposobnost racionalnog propisivanja antibiotika u dentalnoj medicini:
   a) Nedovoljno b) Prosječno c) Iznad prosjeka

12. Koliko u prosjeku propišete antibiotika u radnom tjednu u svojoj praksi:____

13. Praksa primjene antibiotika u dentalnoj medicini i stavovi o antibiotsko rezistenciji:

**Pridržavam se trenutnih smjernica o pravilnom kurativnom i profilaktičkom propisivanju antibiotika u dentalnoj medicini.**	Da	Ne
**Pratim ažuriranje smjernica o pravilnoj primjeni antibiotika u dentalnoj medicini.**	Da	Ne
**Smatram se tijekom dodiplomskog i poslijediplomskog školovanja dovoljno educiranim na temu antibiotika i njihovu primjenu u dentalnoj medicini.**	Da	Ne
**Zainteresiran sam za buduću edukaciju na temu antibiotika i njihove primjene u dentalnoj medicini.**	Da	Ne
**Upoznat s problemom antibiotske rezistencije.**	Da	Ne
**Pri prepisivanju antibiotske terapije uzimam u obzir antibiotsku rezistenciju.**	Da	Ne
**Smatram antibiotsku rezistenciju ozbiljnom prijetnjom javnom zdravlju.**	Da	Ne

**Ponekad propisujem antibiotike na zahtjev pacijenata.**	Da	Ne
**Ponekad propisujem antibiotike kada nisu indicirani iako nisu na zahtjev pacijenta.**	Da	Ne
**Smatram da se antibiotici pretjerano i neselektivno prepisuju u dentalnoj medicini.**	Da	Ne
**Poznajem preporučene doze pojedinih vrsta antibiotika.**	Da	Ne

14. Na temu primjene antibiotika, najviše ste educirani iz kojeg izvora (višestruki izbor):

a) Fakultet
b) Seminari i kongresi
c) Kolege
d) Farmaceuti
e) Internet
f) Stručni članci i znanstveni radovi
g) Ostalo

15. Koji antibiotik birate kao prvi izbor za liječenje dentoalveolarnih infekcija u zdravih pacijenta koji nisu alergični na penicilin (višestruki izbor):

a) Penicilin V - fenoksimetilpenicilin
b) Aminopenicilini
c) Amoksicilin/klavulanska kiselina
d) Tetraciklini
e) Cefalosporini
f) Ertiromicin
g) Klaritromicin
h) Azitromicin
i) Klindamicin
j) Metronidazol
k) Ostalo

16. Koji antibiotik birate za liječenje dentoalveolarnih infekcija u pacijenta koji su alergični na penicilin ili su ga ranije koristili za drugu infekciju (višestruki izbor):

a) Penicilin V - fenoksimetilpenicilin
b) Aminopenicilini
c) Amoksicilin/klavulanska kiselina
d) Tetraciklini
e) Cefalosporini
f) Ertiromicin
g) Klaritromicin
h) Azitromicin
i) Klindamicin
j) Metronidazol
k) Ostalo

17. Koliko dana uobičajeno propisujete antibiotike u liječenju dentoalveolarne infekcije:

a) ≤3 dana b)3-5 dana c) 5-7 dana d) ≥ 7 dana

18. Uz koje grane dentalne medicine vežete najčešće prepisivanje antibiotika u Vašoj ordinaciji (višestruki izbor):

a) Dječja i preventivna dentalna medicina
b) Endodoncija i restaurativna dentalna medicina
c) Oralna kirurgija
d) Oralna medicina
e) Ortodoncija
f) Parodontologija
g) Protetika dentalne medicine

19. Praksa propisivanja antibiotika u kurativne i profilaktičke svrhe za pojedina stanja u medicinski zdravog pacijenta:

**Teški dentofacijalni apscesi, brzo šireći; celulitis i Ludwigova angina**	Da	Ne
**Akutne periapikalne infekcije kod kojih postoji sistemski znakovi upale, povišena temperatura i povećanih limfnih čvorova**	Da	Ne
**Osteomijelitis**	Da	Ne
**Osteonekroza čeljusti povezana s lijekovima sa sekundarnom infekcijom**	Da	Ne
**Perikoronitis**	Da	Ne
**Suha alveola ili lokalizirani osteitis**	Da	Ne
**Akutni sinusitis**	Da	Ne
**Akutni bakterijski sialadenitis**	Da	Ne
**Gingivitis**	Da	Ne
**Nekrotizirajući ulcerativni gingivitis kod kojih postoji sistemski znakovi upale, povišena temperatura i povećanih limfnih čvorova**	Da	Ne
**Parodontni apscesi kod kojih postoji sistemski znakovi upale, povišena temperatura i povećanih limfnih čvorova**	Da	Ne
**Peri-implantatni mukozitis**	Da	Ne
**Peri-implantitis**	Da	Ne
**Postavljanje implantata**	Da	Ne
**Intraoralna koštana augmentacija prije postavljanja implantata**	Da	Ne
**Akutni pulpitis**	Da	Ne
**Avulzija zuba**	Da	Ne
**Oroantralna komunikacija**	Da	Ne
**Ortognatna kirurgija**	Da	Ne

20. Praksa propisivanja antibiotika u profilaktičke svrhe za interventne stomatološke zahvate u medicinski kompromitiraneih pacijenta:

**Prethodni, recidiv ili rekurentni infektivni endokarditis**	Da	Ne
**Protetski srčani zalistak ili materijal**	Da	Ne
**Primatelji transplantacije srca koji razviju srčanu valvulopatiju**	Da	Ne
**Srčani stimulatori, penis, dojke ili intraokularni implantati**	Da	Ne
**Kemoterapija**	Da	Ne
**Radioterapija**	Da	Ne
**Transplantirani organi**	Da	Ne
**HIV**	Da	Ne
**Dijabetes**	Da	Ne
**Renalna dijaliza**	Da	Ne
**Umjetni zglobovi**	Da	Ne
**Uređaji za intravenski pristup (centralne intravenske linije/stalni kateteri koji se koriste za parenteralnu prehranu ili kemoterapiju i kateteri za hemodijalizu)**	Da	Ne
**Autoimuna bolest (npr. juvenilni artritis, sistemski lupus erythematosus)**	Da	Ne

21. Zaokružite odgovor koji smatrate ispravnim:

**Antibiotici se preporučuju samo kao dodatak u konačnom liječenju infekcija, posebice kada postoji povišena temperatura, dokaz sistemskog širenja infekcije te lokalna zahvaćenost limfnih čvorova.**	Točno	Netočno	Ne znam
**Antibiotici se preporučuju uz inciziju, drenažu i uklanjanje uzroka teških dento-alveolarnih infekcija koje se brzo šire.**	Točno	Netočno	Ne znam
**Rutinsko propisivanje klindamicina, cefalosporina ili ko-amoksiklava za dentalne infekcije se ne preporučuje i treba biti izvršeno samo prema uputama specijalista.**	Točno	Netočno	Ne znam
**Antimikrobna sredstva se ne preporučuju za kronične dento-alveolarne infekcije.**	Točno	Netočno	Ne znam
**Penicilin, kao što je fenoksimetilpenicilin ili amoksicilin, prvi je antibiotik izbora za dentoalveolarne infekcije.**	Točno	Netočno	Ne znam
**Antimikrobno sredstvo drugog izbora za dentoalveolarne infekcije je ili metronidazol ili makrolid, npr. klaritromicin.**	Točno	Netočno	Ne znam
**Za antibiotku profilaksu pacijentima se daje amoksicilin 2 g oralno 30-60 min prije zahvata ako stomatološki zahvat uključuje manipulaciju gingivnog tkiva ili periapeksne regije zuba ili perforaciju oralne sluznice.**	Točno	Netočno	Ne znam
**U slučaju alergije na penicilin alternativni antibiotska profilaksa jest Cephalexin 2 g oralno ili azitromicin/klaritomicin 500 mg ili Doxycycline 100 mg oralno.**	Točno	Netočno	Ne znam

22. Jesu li Vam se pacijent javljali s nuspojavama nakon primjena antibiotika u kurativne ili profilaktičke svrhe: a) Da b) Ne

23. Jesu li pacijenti doživjeli neku od sljedećih nuspojava?

**Proljev/bolovi u trbuhu**	Da	Ne
**Mučnina/povraćanje**	Da	Ne
**Alergijske reakcije kože**	Da	Ne
**Žute oči/koža**	Da	Ne
**Glavobolja**	Da	Ne
**Umor/vrtoglavica**	Da	Ne
**Bolovi u mišićima I zglobovima**	Da	Ne
**Utrnulost**	Da	Ne
**Poremećaj spavanja**	Da	Ne
**Gljivične infekcije**	Da	Ne
**Fotoosjetljivost**	Da	Ne
**Problemi s jetrom**	Da	Ne
**Problem s bubrezima**	Da	Ne
**Anafilaksija**	Da	Ne
**Obojenje noktiju i zubi**	Da	Ne

24. Na koje antibiotike ste doživjeli nuspojave među svojim pacijentima:

____

Knowledge and Antibiotic Prescribing Practices among Dental Practitioners from Croatia, Bosnia and Herzegovina, and Serbia (Questionnaire)

1. Gender: a) male b) female
2. Age (years): _____
3. Academic qualification: a) DMD b) MSc c) PhD
4. Specialty:

a) General dentistry b) Endodontics c) Oral surgery d) Oral medicine
e) Pediatric dentistry f) Orthodontics g) Periodontology h) Prosthodontics i) Family Dentistry

5. Practice setting: a) Private practice b) Health center c) Secondary/tertiary care
6. Clinical working experience (years): 1-5 b) 6-10 c) 11-20 d) ≥21
7. Working hours with patients per day: a) ≤6 hours b) >6 sati hours
8. Number of patients per day: a) ≤10 patients b) >10 patients
9. Area of working: a) Urban b) Rural
10. Country of practice: a) Croatia b) Bosnia and Herzegovina c) Serbia

11. Assess your knowledge and ability for rational antibiotic prescribing in dental medicine:
    a) Insufficient b) Average c) Above average

12. On average, how many antibiotics do you prescribe in a working week in your practice: ____

13. Practice of antibiotic use in dental medicine and attitudes towards antibiotic resistance:

**I adhere to current guidelines on curative and prophylactic antibiotic prescribing in dental medicine.**	Yes	No
**I follow updates on the guidelines for proper antibiotic use in dental medicine.**	Yes	No
**During undergraduate and postgraduate training, I am adequately educated on antibiotics and their use in dental medicine.**	Yes	No
**I am interested in future education on antibiotics and their use in dental medicine.**	Yes	No
**I am aware of the issue of antibiotic resistance.**	Yes	No
**I take antibiotic resistance into account when prescribing antibiotic therapy.**	Yes	No
**I consider antibiotic resistance a serious public health threat.**	Yes	No
**I sometimes prescribe antibiotics at the request of patients.**	Yes	No
**I sometimes prescribe antibiotics when they are not indicated, even without patient request.**	Yes	No
**I believe antibiotics are overprescribed and used non-selectively in dental medicine.**	Yes	No
**I am familiar with the recommended dosages of different types of antibiotics.**	Yes	No

14. Source of antibiotic information (multiple answers possible):
a) Dental school b) Seminars and congresses c) Colleagues d) Pharmacists e) Internet
f) Articles and scientific papers

15. A first-choice antibiotic for treating dentoalveolar infections in healthy patients with no allergy to penicillin (multiple answers possible):
a) Penicillin V - Phenoxymethylpenicillin
b) Aminopenicillins
c) Amoxicillin/Clavulanic Acid
d) Tetracyclines
e) Cephalosporins
f) Erythromycin
g) Clarithromycin
h) Azithromycin
i) Clindamycin
j) Metronidazole
k) Other

16. A first-choice antibiotic for treating dentoalveolar infections in healthy patients with a penicillin allergy (multiple answers possible):
a) Penicillin V - Phenoxymethylpenicillin
b) Aminopenicillins
c) Amoxicillin/Clavulanic Acid
d) Tetracyclines
e) Cephalosporins
f) Erythromycin
g) Clarithromycin
h) Azithromycin
i) Clindamycin
j) Metronidazole
k) Other

17. The average duration of prescribed antibiotic therapy for dentoalveolar infection:
a) ≤3 days b)3-5 days c) 5-7 days d) ≥ 7 days

18. The most common procedures that require an antibiotic prescription (multiple answers possible):
a) Pediatric Dentistry b) Endodontics and Restorative Dentistry c) Oral surgery
d) Oral medicine e) Orthodontics f) Periodontology

19. Practice of prescribing antibiotics for curative and prophylactic purposes for specific conditions in medically healthy patients:

**Severe dentofacial abscesses, rapidly spreading; cellulitis and Ludwig's angina**	Yes	No
**Acute periapical infections with systemic signs of inflammation, fever, and swollen lymph nodes**	Yes	No
**Osteomyelitis**	Yes	No
**Medication-related osteonecrosis of the jaw with secondary infection**	Yes	No
**Pericoronitis**	Yes	No
**Dry socket or localized osteitis**	Yes	No
**Acute sinusitis**	Yes	No
**Acute bacterial sialadenitis**	Yes	No
**Gingivitis**	Yes	No
**Necrotizing ulcerative gingivitis with systemic signs of inflammation, fever, and swollen lymph nodes**	Yes	No
**Periodontal abscesses with systemic signs of inflammation, fever, and swollen lymph nodes**	Yes	No
**Peri-implant mucositis**	Yes	No
**Peri-implantitis**	Yes	No
**Implant placement**	Yes	No
**Intraoral bone augmentation before implant placement**	Yes	No
**Acute pulpitis**	Yes	No
**Tooth avulsion**	Yes	No
**Oroantral communication**	Yes	No
**Orthognathic surgery**	Yes	No

20. Practice of prescribing antibiotics for prophylactic purposes in dental interventions for medically compromised patients:

**Previous, recurrent, or relapsing infective endocarditis**	Yes	No
**Prosthetic heart valve or material**	Yes	No
**Heart transplant recipients who develop valvulopathy**	Yes	No
**Pacemakers, penile, breast, or intraocular implants**	Yes	No
**Chemotherapy**	Yes	No
**Radiotherapy**	Yes	No
**Transplanted organs**	Yes	No
**HIV**	Yes	No
**Diabetes**	Yes	No
**Renal dialysis**	Yes	No
**Artificial joints**	Yes	No

**Intravenous access devices (central intravenous lines/permanent catheters used for parenteral nutrition or chemotherapy, and hemodialysis catheters)**	Yes	No
**Autoimmune disease (e.g., juvenile arthritis, systemic lupus erythematosus)**	Yes	No

21. Please choose the correct answer:

**Antibiotics are recommended as an adjunct in the definitive treatment of infections, especially when there is fever, evidence of systemic spread of infection, and local involvement of lymph nodes**	Yes	No	Do not know
**Antibiotics are recommended alongside incision, drainage, and removal of the cause for severe dentoalveolar infections that spread rapidly**	Yes	No	Do not know
**Routine prescription of clindamycin, cephalosporins, or co-amoxiclav for dental infections is not recommended and should only be done based on specialist guidance**	Yes	No	Do not know
**Antimicrobial agents are not recommended for chronic dentoalveolar infections**	Yes	No	Do not know
**Penicillin, such as phenoxymethylpenicillin or amoxicillin, is the first-choice antibiotic for dentoalveolar infections**	Yes	No	Do not know
**The second-choice antimicrobial agent for dentoalveolar infections is either metronidazole or a macrolide, such as clarithromycin**	Yes	No	Do not know
**For antibiotic prophylaxis, patients are given 2 grams of oral amoxicillin 30-60 minutes before the procedure if the dental procedure involves manipulation of gingival tissue or the periapical region of the tooth or perforation of the oral mucosa**	Yes	No	Do not know
**In case of penicillin allergy, alternative antibiotic prophylaxis includes Cephalexin 2 grams orally, Azithromycin/Clarithromycin 500 mg orally, or Doxycycline 100 mg orally**	Yes	No	Do not know

22. Have patients reported adverse effects to you after the use of antibiotics for curative or prophylactic purposes: a) Yes b) No

23. Have patients experienced any of the following adverse effects?

**Diarrhea/abdominal pain**	Yes	No

**Nausea/vomiting**	Yes	No
**Skin allergic reactions**	Yes	No
**Yellowing of the eyes/skin**	Yes	No
**Headache**	Yes	No
**Fatigue/dizziness**	Yes	No
**Muscle and joint pain**	Yes	No
**Numbness**	Yes	No
**Sleep disturbances**	Yes	No
**Fungal infections**	Yes	No
**Photosensitivity**	Yes	No
**Liver problems**	Yes	No
**Kidney problems**	Yes	No
**Anaphylaxis**	Yes	No
**Discoloration of nails and teeth**	Yes	No

24. Which antibiotics have your patients experienced adverse effects from? ____
